# Effectiveness of dilution ventilation in mitigating occupational exposure to volatile organic compounds (VOCs) at the breathing zone of nail technicians: a simulation study

**DOI:** 10.1038/s41598-025-33777-y

**Published:** 2026-01-03

**Authors:** Samaneh Salari, Sahar Fazeli-Tabar, Ali Karimi, Marjan Fazlali

**Affiliations:** 1 Department of Occupational Health Engineering, School of Health, Tehran University of Medical Sciences, Poursina St, Keshavarz Blv., Tehran, Iran; 2 Department of Tourism, University of Science and Culture, Tehran, Iran

**Keywords:** VOC, Nail salon, Dilution ventilation, ACH, Simulation, Occupational exposure, Environmental sciences, Health occupations, Risk factors

## Abstract

Volatile Organic Compounds (VOCs) from nail products pose potential health risks to technicians and clients. The present study investigates the exposure of nail technicians to VOC and assesses the effectiveness of dilution ventilation in mitigating these exposures. A test chamber was configured to simulate a nail salon environment, where three common activities were performed: applying nail polish, removing nail extensions, and nail extension application. A MultiRae Lite gas meter was used to monitor VOC concentrations in the technicians’ breathing zones and the ambient environment under both non-ventilated and dilution-ventilated conditions. The average VOC concentrations in the technicians’ breathing zones were measured at 8.3 (± 8.576) ppm, 116.2 (± 120.04) ppm, and 29.73 (± 7.876) ppm during nail polish application, nail extension removal, and nail extension application, respectively (*n* = 30 measured over 15 min). For the same activities, the average ambient VOC concentrations in the non-ventilated chamber were 25.03 (± 13.006) ppm, 192.17 (± 114.900) ppm, and 40.90 (± 30.891) ppm. These concentrations were significantly lowered by dilution ventilation to 0.93 (± 0.179) ppm, 12.6 (± 5.21) ppm, and 2.63 (± 0.812) ppm. For all three activities, a t-test verified a statistically significant drop in VOC concentration with dilution ventilation (*p* < 0.001). Additionally, the study discovered that VOC emissions could be reduced by over 65% at an air change rate (ACH) of 10 per hour. The results indicate that dilution ventilation can significantly decrease VOC exposure; nonetheless, the study concludes that it is insufficient as the sole control strategy in nail salons because concentrations failed to reach the limit levels, even at the highest tested ACH (100 ACH). This research provides a new scientific perspective on ventilation as a means of controlling occupational exposure to VOC in nail salons.

## Introduction

In nail salons, the products used for manicures, pedicures, nail designs, and nail extension release various pollutants. Numerous studies have demonstrated that volatile organic compounds (VOC), such as methyl methacrylate, acetone, ethyl acetate, benzene, toluene, and formaldehyde, constitute major air pollutants in nail salons^1–4^. Nail technicians exposed to these substances may experience short-term symptoms such as headaches and neurological issues, as well as long-term symptoms such as respiratory, skin, and eye irritation^5^, damage to the liver and kidney system^6^, and reproductive disorders^7,8^.

Moreover, nail beauty services as a part of the service industry, have been recognized by NIOSH’s National Occupational Research Program as an area requiring investigation and intervention to reduce occupational diseases and injuries^9,10^. The ACGIH and OSHA have specific instructions for each volatile organic compound, which report the Threshold Limit Value (TLV) and Permissible Exposure Limit (PEL), respectively^11,12^. For total volatile organic compounds (TVOC), the USEPA established a maximum allowable air concentration standard of less than 0.2 mg/m^3^^13^.

Despite the exposure to VOC posing serious threats, regulations protecting beauty technicians are often inadequate. Many nail technicians are exposed to VOC for more than eight hours every day^2^. On the other hand, numerous studies have shown that many nail salons have inadequate ventilation^7,14–16^, even the presence of high VOC concentrations. Meanwhile, over 60% of nail technicians fail to put on any personal protective equipment^17,18^.

Assessing exposure to VOC and implementing effective ventilation in nail salons is essential. Determining the rate of VOC released during various nail salon activities is a useful step in detecting and evaluating health concerns and can assist with control measures implementation^19^. On the other hand, it is necessary to verify proper ventilation for the nail salon^20^. VOC exposures can be decreased by improving ventilation to satisfy minimal outdoor air delivery requirements^21^.

There are several restrictions on the exposure assessment of nail salons, such as the small and confined workplaces, the existence of interventions, and the restricted accessibility to the technicians^22–25^. Therefore, in the present study, three routine nail beauty activities including applying nail polish, removing nail extension, and nail extension were simulated in a test chamber. Technician exposure to all three activities was evaluated in non-ventilated and with dilution ventilation. To our knowledge, this is the first study to quantitatively investigate the effectiveness of dilution ventilation in mitigating VOC exposure at the breathing zone of nail technicians under controlled laboratory settings, including extreme air change rates. The results of the present study are expected to create a new scientific perspective in the field of ventilation concerning occupational VOC exposure in nail salons.

## Methods and materials

### Instrument and materials

The materials and instruments needed for a nail salon simulation were selected based on those commonly utilized in the Iranian market. These materials include nail glue, liquid, acrylic powder, Plexiglas nail tips, pure acetone with the formula (Bitabhdis Company, Tehran, Iran), three comparable solvent-based nail polish brands, and primer/anti-fungal agent (dehydrator) from the NBI^®^ brand.

A MultiRae lite gas meter, Wireless - MAB3-A2C112E-420 model (RAE Co, USA) was used for real-time measurement of VOC. This device employs a Photoionization Detector (PID) sensor as its measurement principle for VOC. The PID uses a 10.6 eV UV lamp, which is necessary for ionizing the VOC into detectable charged particles. This sensor is configured to measure Total Volatile Organic Compounds (TVOC) in the range of 0 to 1,000 ppm with a resolution of 1 ppm. PID readings are reported as TVOC in isobutylene equivalents.

Comprehensive Quality Assurance/Quality Control (QA/QC) procedures were implemented to ensure data accuracy and reliability:

#### Calibration gas and frequency

The instrument was span-calibrated automatically with the AutoRAE 2 using 100 PPM isobutylene standard gas prior to the start of the experimental period. Daily bump tests were performed to confirm sensor functionality, and zero checks were performed weekly by sampling clean air. Calibration drift checks were also performed weekly.

#### Humidity/temperature compensation

Environmental conditions were monitored throughout the study. The monitoring took place at ambient temperatures ranging from 22 to 25 °C and relative humidity (RH) between 40% and 50%. The MultiRAE Lite unit features internal temperature and humidity compensation to minimize their effect on the PID sensor readings; consequently, no external corrections were applied.

PID within the MultiRAE Lite instrument was calibrated using isobutylene standard gas. was calibrated with isobutylene standard gas. Consequently, all reported TVOC concentrations are presented as isobutylene equivalents in ppm. Consequently, VOC reduction claims are based on the comparative reduction of the IBE signal, and do not account for component-specific response factors.

In addition, a digital anemometer, Model PROVA AVM05 was used. The measurement range for Wind Velocity is 0.0 to 45.0 m/s, with accuracy ± 3% ± 0.1dgts (resolution = 0.1).

### Measurement and evaluation of VOC

**Fig. 1:**
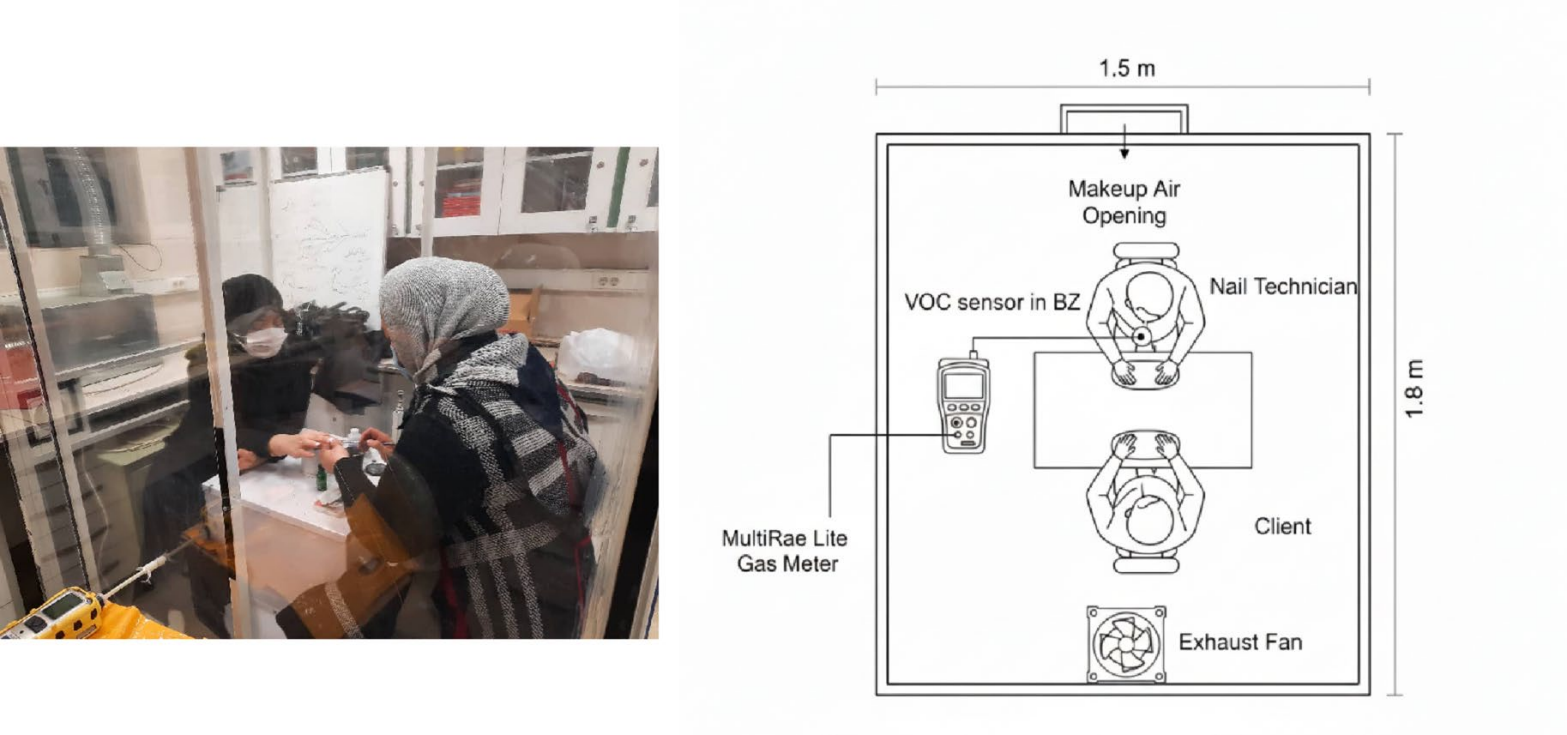
Assessing VOC exposure during nail technician activities. Left: Experimental simulation for measuring VOC in test chamber. Right: Schematic layout of the test chamber (generated with AI assistance using ChatGPT).

The present study was conducted to evaluate the emission of VOC resulting from nail art in the test chamber of the medical sciences university, as shown in Fig. 1. A test chamber proven to be airtight was utilized to simulate major nail beauty activities including applying nail polish, removing nail extensions, and nail extension. The activities simulated are identical to those performed in a nail salon at each stage. At the stage of using acetone and liquid materials, the lid of the solution was closed immediately after use. Furthermore, during the test chamber sampling process, highly contaminated disposable cotton and napkins were taken out of the test chamber to minimize the number of pollutants that evaporated from them.

In the test chamber, VOC emission was monitored under two different conditions: dilution ventilation and non-ventilation. To perform this task, the nail technician performed each activity on the candidate for 10 to 15 min under specific conditions. Meanwhile, the concentration of VOC in the breathing zone (BZ) was measured using a gas meter during each activity, with samples taken every 30 s. The VOC concentration in BZ and the amount emitted in the Test Chamber (TC) have been measured.

The emission rate (E) was calculated using the time-resolved VOC concentration data (C(t)) obtained from the 30-second sampling interval, based on the principle of mass-balance kinetics within the test chamber. In the non-ventilated condition (ACH = 0), assuming a negligible decay rate ($\:k\approx\:0)$, the mass-balance equation simplifies to a linear accumulation model ($\:\frac{dC}{dt}=\frac{E}{V}$)^26^.

The VOC emission rate was inferred by performing a linear regression fit on the $\:C\left(t\right)$ time series for each activity.

The slope of the regression line ($\:\frac{dC}{dt}$), reported in ppm/min, was used to calculate the emission rate in $\:\mu\:g/min$ using the following relationship:

1 $$\:{E}_{(\mu\:g/min)}={\left(\frac{dC}{dt}\right)}_{ppm/min}\times\:{V}_{\left({m}^{3}\right)}\times\:\frac{{MW}_{avg}\left(g/mol\:\right)}{24.45}\times\:{10}^{3}$$

where V is the chamber volume, $\:{MW}_{avg}$ is the average molecular weight, and the factor $\:{10}^{3}$ converts grams to micrograms.

### Simulation of dilution ventilation

In the present study, the efficiency of dilution ventilation was investigated for reducing the level of VOC emitted. For this purpose, an exhaust fan and a makeup air opening were mounted in the test chamber. To optimize the efficiency of dilution ventilation and minimize contaminant exposure in the BZ, the exhaust fan was situated close to the exposed technician, and the makeup air opening was positioned behind the technician (Fig. 1). The Air Changes per Hour (ACH), also known as the air exchange rate, is defined as the number of times the entire air volume in the test chamber is completely withdrawn and refilled in an hour. In the present study, ACH of 0, 5, 10, 20, 30, 40, and, 100 $\:{h}^{-1}$ were investigated in the dilution ventilation. The selected ACH range (0, 5, 10, 20, 30, 40, and 100) was strategically chosen to cover standard regulatory requirements 5 to 20 ACH), enhanced engineering controls (30 to 40 ACH), and aggressive control scenarios (100 ACH). The aggressive control rates have been tested to demonstrate that even the highest dilution ventilation rates cannot provide comprehensive control for nail salon. This range provides a comprehensive performance benchmark for high-emission environments, consistent with previous studies on ventilation effectiveness in industrial and laboratory settings^27,28^.

The required volumetric flow rate ($\:{\mathrm{Q}}_{\mathrm{r}\mathrm{e}\mathrm{q}\mathrm{u}\mathrm{i}\mathrm{r}\mathrm{e}\mathrm{d}}$) for each ACH was calculated using the chamber volume (V), and the necessary face velocity (v) to achieve that flow was determined using the opening area (A). The required airflow was determined during the different tests using the following equations^29^:

2 $$\:\mathrm{A}\mathrm{C}\mathrm{H}=\frac{60{\mathrm{Q}}_{\mathrm{r}\mathrm{e}\mathrm{q}\mathrm{u}\mathrm{i}\mathrm{r}\mathrm{e}\mathrm{d}}}{{\mathrm{V}}_{\mathrm{r}\mathrm{o}\mathrm{o}\mathrm{m}}}$$,

3 $$\:\mathrm{v}=\raisebox{1ex}{${\mathrm{Q}}_{\mathrm{r}\mathrm{e}\mathrm{q}\mathrm{u}\mathrm{i}\mathrm{r}\mathrm{e}\mathrm{d}}$}\!\left/\:\!\raisebox{-1ex}{$\mathrm{A}$}\right.$$,

where ACH: Air changes per hour ($\:{h}^{-1}$). $\:\mathrm{v}$: Face velocity (fpm). $\:{\mathrm{V}}_{\mathrm{r}\mathrm{o}\mathrm{o}\mathrm{m}}$: volume of the test chamber (76.08 $\:{\mathrm{f}\mathrm{t}}^{3}$). $\:{\mathrm{Q}}_{\mathrm{r}\mathrm{e}\mathrm{q}\mathrm{u}\mathrm{i}\mathrm{r}\mathrm{e}\mathrm{d}}$ : required volumetric flow rate (cfm). A: Opening area (0.10 ft^2^) (The diameter of the inlet/outlet opening was 0.36 ft).

The calculated dilution airflow rate for 10 ACH is 12.68 cfm. A damper was used in conjunction with a digital anemometer to establish and maintain the required face velocity (v) for the exhaust fan.

For example (Setting Velocity for 10 ACH).

To achieve 10 ACH, the required flow rate (Q) and velocity (v) are calculated:

$$\:Q=76.08\:{ft}^{2}\times\:\frac{{10\:h}^{-1}}{60\:min/hr}=12.68\:cfm$$

$$\:v\:=\frac{12.68\:cfm}{0.10\:{ft}^{2}\:}=126.8\:fpm$$

The corresponding required face velocities for 10, 20, 30, and 40 ACH were verified to be 126.8, 253.6, 380.4, and 507.2 fpm respectively. Three activities were carried out in the test chamber for 15 min while setting the required velocity for each air exchange rate. The measured VOC concentration was recorded every 30 s.

## Result

VOC concentrations were measured under two conditions—non-ventilation and dilution ventilation—during the simulation of three crucial nail art activities involving a nail technician in the laboratory test chamber. Findings from these two conditions, along with the results on the impact of the air exchange rate, will be reported in the following sections.

### concentration of VOC in a non-ventilated test chamber

**Fig. 2:**
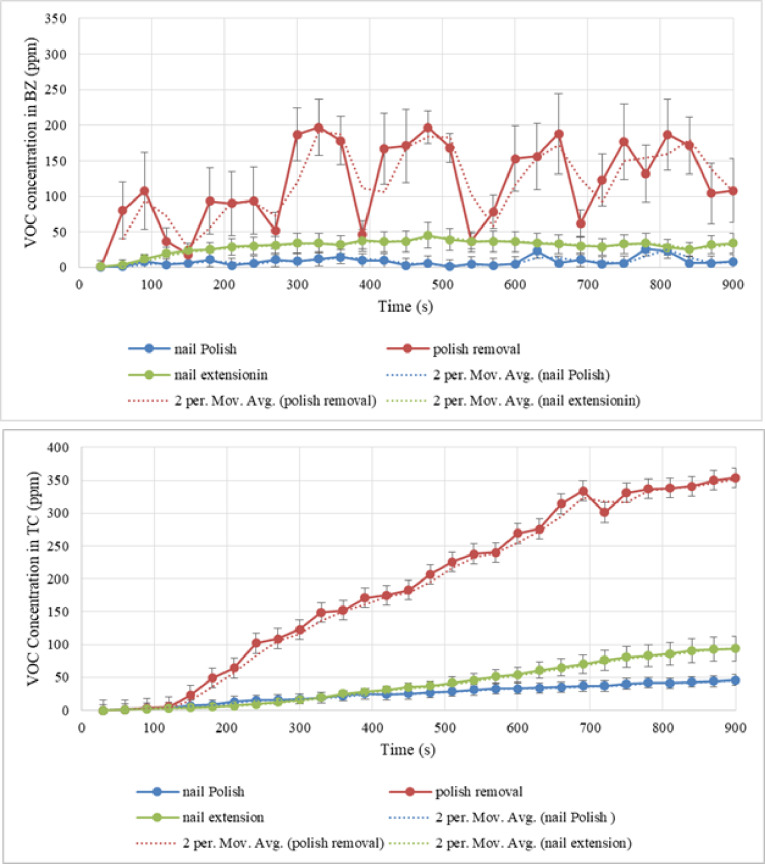
VOC concentration in the breathing zone (VOC in BZ) during activities (Top graph) and VOC concentration in the non-ventilated test chamber (VOC in TC) during three activities (Bottom graph).

At the end of the 15-minute nail polish operation in the non-ventilated test chamber, the MultiRae lite gas meter recorded a VOC concentration of 43 ppm. The VOC mass concentration was estimated to be 31.08 $$\:\raisebox{1ex}{$mg$}\!\left/\:\!\raisebox{-1ex}{${m}^{3}$}\right.$$ (15 min) following the chemical characteristics of the materials used in the simulation (2.38 conversion coefficient). The TVOC mass released was estimated to be 67.13 mg considering the chamber’s volume, corresponding to an average emission rate of 4.475 mg per minute. Figure 2 illustrates VOC released in the breathing zone for all three activities under the non-ventilated.

In the same way, the quantity of VOC released during the two activities of removing and applying nail extensions was estimated. The gas meter detected VOC concentrations were detected of 341 ppm and 108 ppm at the end of the 15-min period during nail extension removal and nail extension, respectively. In this regard, during removing nail extension, an average emission rate of 116.89 $$\:\raisebox{1ex}{$mg$}\!\left/\:\!\raisebox{-1ex}{${m}^{3}$}\right.$$ was generated. This is while the operation of nail extension produced 37.03 mg/min on average. The trend of VOC emission during these activities over the course of the breathing zone sampling is depicted in the bottom graph of Fig. 2. The significant spike and scatter observed in the breathing zone concentration, particularly during nail extension removal (due to the large volume of acetone use), are attributed to the high emission rate and the proximity of the concentrated VOC source to the sensor intake. As shown in the top graph of Fig. 2, the activity of removing nail extensions in the non-ventilated test chamber resulted in the highest VOC emission.

### Determining the VOC concentration in the test chamber with dilution ventilation

In the test chamber, an exhaust fan and a makeup air opening were implemented to evaluate the efficiency of dilution ventilation in reducing the level of contaminants emitted. Under dilution ventilation conditions, the average VOC with standard deviation (SD) for the three operations—applying nail polish, removing nail extensions, and nail extension—were found to be 0.93 (± 0.179) ppm, 12.6 (± 5.21) ppm, and 2.63 (± 0.812) ppm, respectively.

**Fig. 3:**
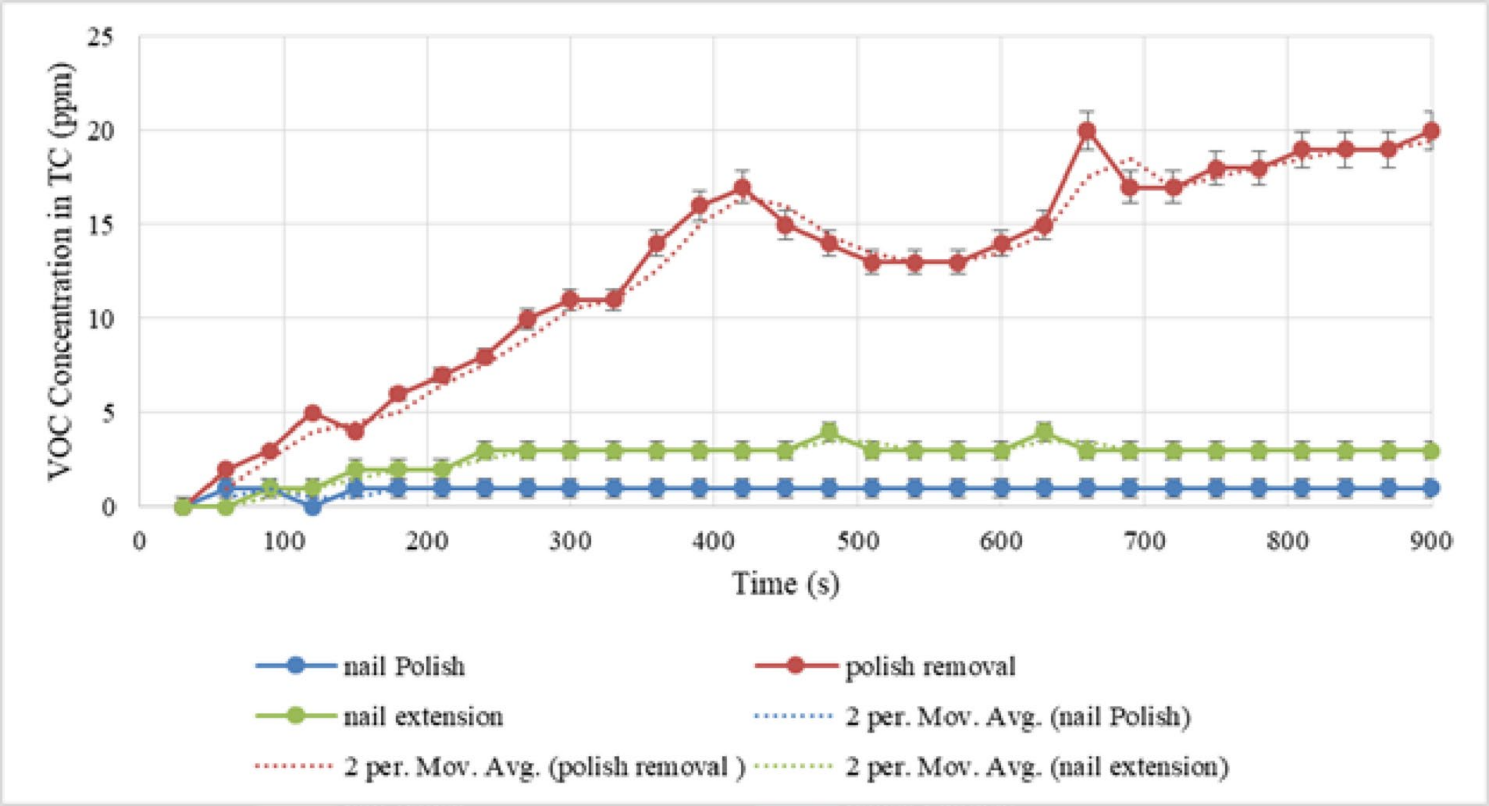
VOC concentration in the dilution ventilation of the test chamber (VOC in TC) during three activities.

The VOC emission during activities in the test chamber with dilution ventilation is shown in Fig. 3. A t-test with a 95% confidence level revealed a statistically significant difference (p-value < 0.001) between the VOC concentrations in dilution ventilation and non-ventilation across all three activities.

### Evaluating ACH in dilution ventilation

The ACH, also known as the air exchange rate, is defined as the number of times the entire volume of air in a room is completely withdrawn and replenished in one hour. In this work, ACH in dilution ventilation for VOC vapor reduction was analyzed using a makeup air opening and exhaust fan mounted in the test chamber. According to the ACH formula (Eq. 2 in “Simulation of dilution ventilation”), a speed of 126.8 fpm, 253.6 fpm, 380.4 fpm, and 507.2 fpm were required for 10, 20, 30, and 40 ACH, respectively. A makeup air damper was used to control the fan speed to achieve the required face velocity. The speed of the air entering the test chamber was measured using an anemometer.

**Fig. 4:**
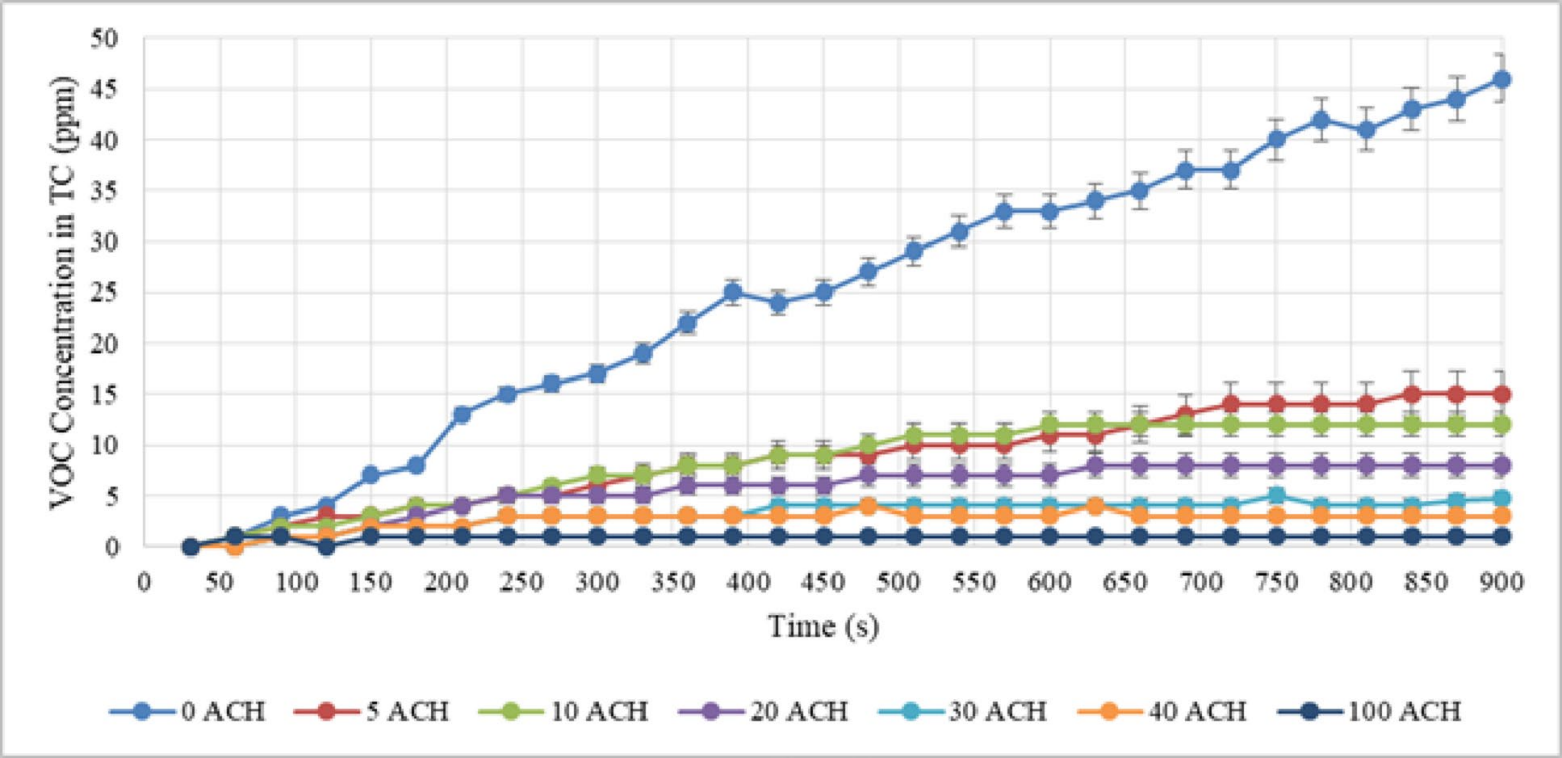
VOC concentration in test chamber with different ACH during applying nail polish.

**Fig. 5:**
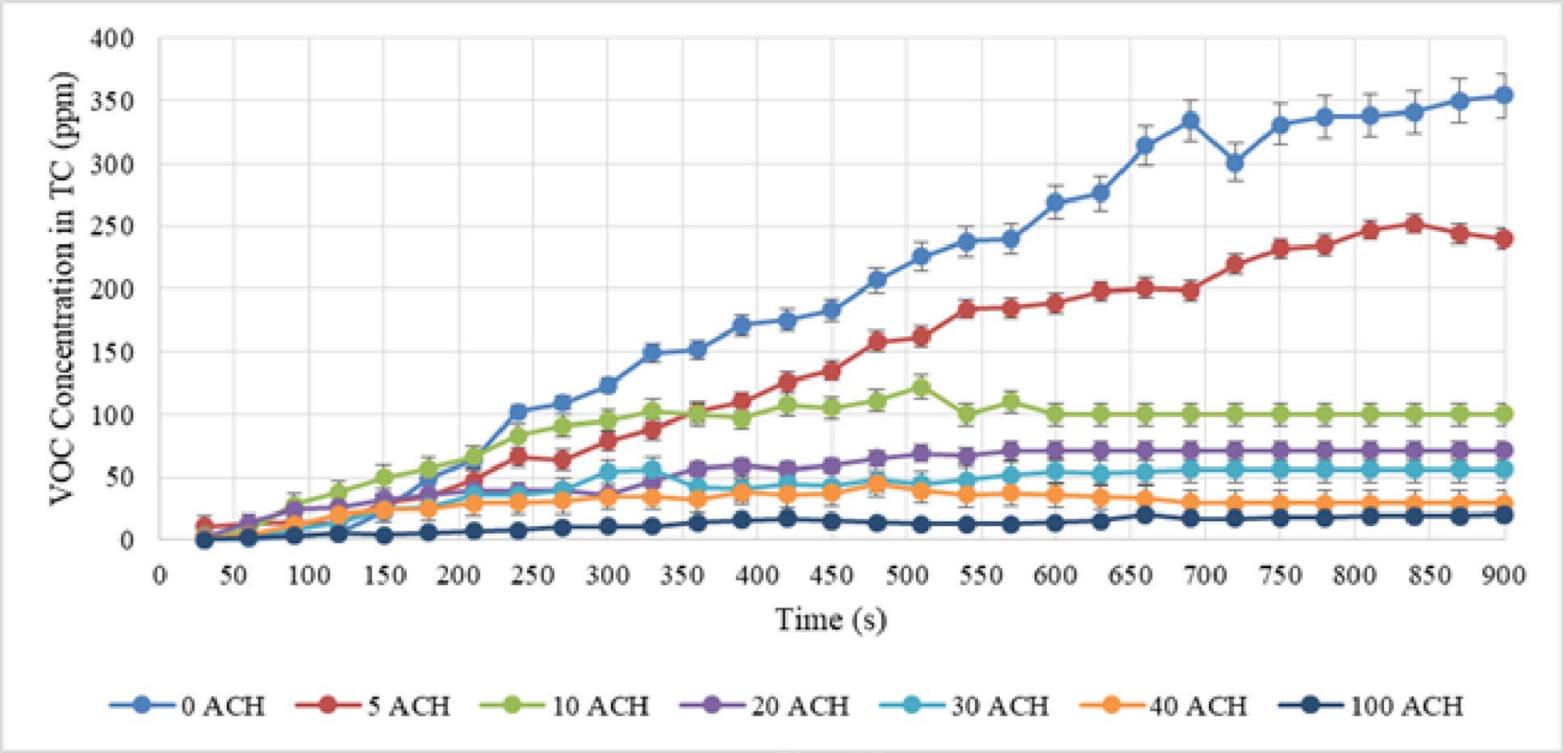
VOC concentration in test chamber with different ACH during removing nail extension.

**Fig. 6:**
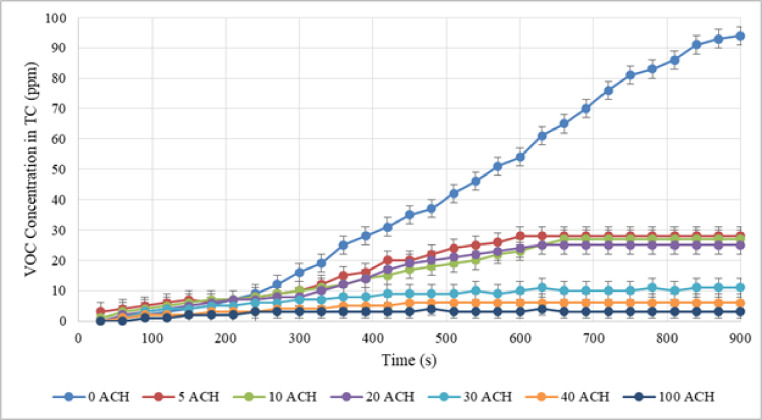
VOC concentration in test chamber with different ACH during nail extension.

After setting the desired speed, all three activities were performed for 15 min by the nail technician. Additionally, the detected concentration of VOC was recorded every 30 s. Figures 4, 5, and 6 illustrate the VOC concentration over time during applying nail polish, removing nail extensions, and nail extension, respectively.

Permissible limits for VOC vary significantly between international health and occupational standards. The World Health Organization (WHO) classifies air quality with stringent health-based guidelines, setting a high threshold for total VOC (TVOC) at < 0.61 ppm, (for best air quality: 0–0.05 ppm). Conversely, the LEED (Leadership in Energy and Environmental Design) Green Building Rating System focuses on healthy building design, setting the TVOC limit for its scoring function at less than 500 $$\:\raisebox{1ex}{$\mu\:g$}\!\left/\:\!\raisebox{-1ex}{${m}^{3}$}\right.$$ (approximately 0.133 ppm). Meanwhile, the Occupational Safety and Health Administration (OSHA) primarily uses a qualitative approach, categorizing VOC levels in non-industrial settings based on AQI (Air Quality Index) bands, defining concentrations up to 100 $$\:\raisebox{1ex}{$\mu\:g$}\!\left/\:\!\raisebox{-1ex}{${m}^{3}$}\right.$$ (approximately 0.027 ppm) as good air quality. Table 1 reveals that the VOC concentration (mean$$\:\pm\:$$SD), measured from 30 intervals over the 900-second activity period, demonstrated that dilution ventilation failed to reduce VOC concentration levels below the stringent permissible limits established by organizations like WHO and LEED.

**Table 1:** VOC concentration in test chamber (ppm) across different ACH (*n* = 30).

	0 ACH (non-ventilated)	5 ACH	10 ACH	20 ACH	30 ACH	40 ACH	100 ACH
Applying nail polish	25.037 ± 13.006	8.7 ± 8.99	8.33 ± 8.611	5.67 ± 5.855	3.208 ± 3.315	2.63 ± 2.721	0.93 ± 0.964
Removing nail extension	192.17 ± 114.900	135.96 ± 40.13	85.83 ± 88.594	54 ± 55.733	41.26 ± 42.642	29.33 ± 30.278	3.33 ± 3.444
Nail extension	40.90 ± 30.891	18.43 ± 18.948	16.63 ± 17.154	16.07 ± 16.602	7.80 ± 8.060	4.63 ± 4.788	0.934 ± 0.964

## Discussion

During nail polish application, nail extension removal, and nail extension, the technician’s breathing zone exhibited an average VOC emission rate of 4.475 mg/min, 116.86 mg/min, and 37.03 mg/min, respectively. These results related to the VOC concentration in the breathing zone showed that technicians are exposed to a significant amount of VOC during all three activities. The use of pure acetone to remove nail polish resulted in the highest amount of VOC emission when compared to other activities, as Fig. 2 illustrates.

In one study, after ten minutes of simulated conditions with no ventilation, an average of 50 to 330 ppm of VOC was measured. Similar to our study, VOC amounts were significantly high^30^. In contrast, Fig. 2 displays the lowest VOC emission dispersion for nail polish application. The maximum level of VOC emissions (15 min) in the breathing zone was observed in relation to applying nail polish (43 ppm), nail extension removal (341 ppm), and nail extension (108 ppm).

Nevertheless, there was a nonlinear increase in the breathing zone’s VOC release rate. However, the VOC emission rate in the test chamber’s ambient linearly increased during all three activities. There were higher VOC emission levels during the nail extension removal in the ambient of the test chamber and breathing zones. These results are expected because this activity exclusively employed 100% pure acetone.

Acetone and isopropyl alcohol, which are used to remove nail extensions, were shown to be responsible for at least 65% of the total VOC concentrations found in a study of nail salons in Los Angeles^31^. Isopropyl alcohol is also a solvent in nail polishes. Methyl methacrylate, which is prohibited by the US Food and Drug Administration (FDA) in nail products, was also found in the liquid that is used for nail extensions^31^. On the other hand, the average VOC concentration for nail polish application, nail extension removal, and nail extension application was found to be 25.03 (± 13.006) ppm, 192.17 (± 114.900) ppm, and 40.90 (± 30.891) ppm, respectively, in the ambient TVOC monitoring of the test chamber.

Zhong et al. estimated the headspace concentration of TVOC in nail polish at 211 ppm and nail extension at 47 ppm using monomer powder^16^. Similar to our study, nail polish released more concentration of VOC than nail extension. In addition, the concentration of TVOC in both activities was higher than the permissible limits. Due to the proximity of the technician to the subject, the VOC measurement in the breathing zone was higher than the VOC concentration in the test chamber, in the present study.

Furthermore, Zhong et al.^16^ indicate that TVOC in personal exposure is approximately twice the concentrations detected in the nail salon environment. In the present study, materials that are commonly used in Iran nail salons have been investigated. However, the VOC concentration may also change when nail cosmetics vary^16^.

According to numerous studies conducted in different countries, nail salon personnel are exposed to high levels of VOC^14,20,21^. Therefore, it is necessary to implement proper ventilation for nail salon^20^. TVOC exposures can be decreased by improving nail salons ventilation to satisfy minimal outdoor air delivery requirements^21^. In the present study, VOC levels were monitored for 15 min after exhaust and input fans were installed. This was done to analyze the impact of dilution ventilation.

The results show that despite 5 ACH during nail polish activity, which seems to be a small amount, it reduces the concentration of VOC by about 65%. This is a considerable amount. Therefore, using a dilution ventilation system even at the lowest feasible ACH can be effective. Additionally, air change rates can accomplish practical ventilation protocols in school bus, according to the results of an investigation into control strategies for dilution ventilation in school buses to mitigate the COVID-19 pandemic. The recommended ACHs in the present study varied from 6 to 12 ACH^32^. The average concentration of VOC in polish removal at 0, 5, 10, 20, 30, 40, and 100 ACH in dilution ventilation were estimated as shown in Table 1.

The results reveal that 29% of the pollution was decreased with five ACH due to the high amount of VOC generated during this activity, whereas around 55% of the pollution was reduced with ten ACH. Due to the high amount of VOC generated during the nail extension removal activity, a higher ACH would be required to minimize pollution.

The average VOC released in nail extension was 40.90, 18.43, 16.63, 16.07, 7.80, 4.63, and 3.33 at 0, 5, 10, 20, 30, 40, and a maximum of 100 ACH in dilution ventilation. In this activity, at 5 ACH about 66% of VOC concentration was discharged due to dilution ventilation.

On the other hand, to reduce chemical exposure such as volatile organic compounds, material safety data sheets may recommend changing the air in the workplace ten times or more each hour. Each activity was tested using a separate two-variable t-test to determine the contribution of dilution ventilation to the reduction of VOC compared to non-ventilation. The test results were significant for all three activities (P-value$\:<$ 0.001). The reliability of the comparative analysis was ensured by rigorously verifying the underlying assumptions for the two-variable t-test. Normality of the VOC concentration data was assessed in both the ventilated and non-ventilated conditions using the Shapiro-Wilk test (*p* > 0.05). Furthermore, the homogeneity of variances across the comparison groups was confirmed using Levene’s test (*p* > 0.05). The data successfully satisfied these parametric assumptions, thereby validating the application of the t-test and supporting the robustness of the reported P-values. As a result, dilution ventilation was more effective than non-ventilation in lowering the level of VOC. Furthermore, VOC concentration has decreased in all activities with an increase in air changes per hour. But, even with 100 times air changes per hour, the average concentration of VOC could not be reduced to a level below the permissible limit in any of the activities (e.g., the WHO threshold for temporary exposure of 0.61 ppm). In dilution ventilation, more than 100 air changes per hour might not be cost-effective.

Therefore, it can be claimed that although dilution ventilation can effectively lower exposure, it is insufficient to lower concentrations below occupational exposure limits under typical nail salon conditions. It is necessary to study other types of air pollution control systems that are effective for volatile organic compounds. For example, Lamplugh et al. investigated the removal of VOC emissions from common nail care products using three adsorbents. Lamplugh et al.^33^ suggests that by improving active flow, activated carbon can serve as an effective component of nail salon ventilation systems. On the other hand, a study demonstrated the effectiveness of general exhaust ventilation to reduce TVOC exposure in New York nail salons. It has also been suggested in several studies that further reductions may be achieved by installing LEV systems, which improve by capturing pollutants released near technicians and customers during nail services^14^.

## Limitations and future directions

The present study provides valuable insights into the effectiveness of dilution ventilation in mitigating volatile organic compound (VOC) exposure; however, it is not without its limitations. First, this research was conducted in a controlled laboratory environment using a simulated chamber. While this approach allowed for precise measurements, it may not fully capture the complexities of a real-world nail salon, which can be affected by various factors such as salon layout, customer density, and inconsistent use of personal protective equipment.

Second, the scope of the present study was limited to investigating dilution ventilation and a specific set of commonly used products. Our findings, therefore, cannot be generalized to other control strategies or the full range of chemical products available on the market.

Based on these limitations, we suggest several avenues for future research. Exploring the efficacy of alternative control methods, such as local exhaust ventilation (LEV) or the use of novel absorbent materials, is essential. We also recommend that future studies compare different ventilation methods to determine the most effective and cost-efficient solutions for nail salons.

Finally, our findings highlight a broader issue: the lack of standardized procedures for assessing compliance with ventilation guidelines in nail salons. We propose that establishing clear protocols for initial air quality measurements in salons would be a crucial step toward ensuring public health objectives are met and occupational hazards for both staff and clients are effectively reduced.

## Conclusion

Based on our findings, various strategies are required to reduce the exposure of nail technicians. These include improving ventilation in nail salons, replacing or removing high-toxicity chemicals, and using personal protective equipment as directed.

This research demonstrated that the concentration of VOC in the breathing zone of nail technicians and the surroundings of the test chamber was often significantly higher than the acceptable level. Our results also indicated that personal exposure in the breathing zone is higher due to the proximity of the technician to cosmetic materials and products. It was found that VOC are released more during nail extension removal than in other nail beauty activities.

In the present study, dilution ventilation at different ACH was investigated. The results showed that nail salons could benefit from dilution ventilation. However, it is important to note that based on our findings, this method alone may not be a sufficient control strategy to eliminate VOC, as the observed decrease did not consistently reach the limit levels.

## Data Availability

All data generated or analyzed during present study are included in this published article.
